# Beyond Antibodies in Post-Transplant FSGS: New Answers or Recurrent Questions?

**DOI:** 10.3389/ti.2025.15032

**Published:** 2025-09-10

**Authors:** João Venda, Andreia Henriques, Pedro Fragoso, Clara Pardinhas, Maria Marques, Luís Rodrigues, Rita Leal, Lídia Santos, Catarina Romãozinho, Rui Alves, Arnaldo Figueiredo

**Affiliations:** ^1^ Department of Nephrology, Hospitais da Universidade de Coimbra, Unidade Local de Saúde de Coimbra, Coimbra, Portugal; ^2^ Faculty of Medicine, University of Coimbra, Coimbra, Portugal; ^3^ Department of Urology and Kidney Transplantation, Hospitais da Universidade de Coimbra, Unidade Local de Saúde de Coimbra, Coimbra, Portugal

**Keywords:** FSGS recurrence, glomerulonephritis, immunosuppression, genetics, complement

Dear Editors,

More than fifty years have passed since Hoyer et al. first described recurrent focal segmental glomerulosclerosis (rFSGS) in kidney transplant recipients. Yet, despite significant advances in understanding this disease, graft survival remains alarmingly poor [1, 2]. Circulating permeability factors, particularly anti-nephrin antibodies, have long been implicated in primary rFSGS. A recent study by Batal et al. marked a significant breakthrough, demonstrating 100% specificity of this marker in recurrent cases. However, they also found that 62% of patients who developed rFSGS, did not have anti-nephrin antibody titers and lacked IgG deposits colocalizing with nephrin. This low sensitivity is a reminder that other, yet unidentified, pathogenic factors may contribute to recurrence, and the search for additional culprits is far from over. However, implementing large-scale studies is difficult due to the rarity of rFSGS cases. Moreover, the absence of standardized pathophysiology-based criteria leads to the grouping of different FSGS subtypes — each with distinct clinical, management, and prognostic features - contributing to methodological variability and inconsistent classification across studies. Addressing this heterogeneity rigorously is crucial for improving the accuracy of clinical assessments and the effectiveness of therapeutic interventions.

We have retrospectively reviewed primary rFSGS cases in our institution from January 2010 to December 2020. We included only native kidney biopsy-confirmed cases of FSGS or minimal change disease (MCD), that developed rapid-onset nephrotic syndrome post-transplant. Of the 1,372 kidney transplants performed, six allograft recipients (five males and one female) met these inclusion criteria (Supplementary Figure S1). The baseline and clinical characteristics of these patients are detailed in Table 1. Three had childhood-onset native kidney disease and only one was initially steroid-sensitive. Genetic testing was performed in four patients, and a TRPC6 gene variant was identified in one. Time from the initial diagnosis to the initiation of renal replacement therapy varied widely, ranging from 2 to 19 years. All six patients experienced recurrence within the first month post-KTx with a mean proteinuria of 11 g. Three patients received induction therapy with Basiliximab, three received thymoglobulin, and all were maintained on a triple regimen with steroids, tacrolimus, and anti-metabolite. Upon recurrence, plasmapheresis was promptly initiated in four patients. Three patients achieved complete remission after a median of 81 days. The remaining three patients, unresponsive to initial therapies, also showed a limited response to alternative modalities - additional dose of rituximab (N = 1) and adrenocorticotropic hormone (N = 1) - and progressed to graft loss.

**TABLE 1:** Baseline and clinical characteristics of the study group.

Patient number id	1	2	3	4	5	6
Gender	Male	Male	Male	Male	Female	Male
Age at disease presentation (years)	2	1	5	30	18	45
Native kidney histological findings	FSGS	FSGS	FSGS	FSGS	FSGS	MCD
Initial steroid sensitivity	Steroid-resistant	Steroid-sensitive	Steroid-resistant	Steroid-resistant	Steroid-resistant	Steroid-resistant
Genetic testing	Positive TRPC6 gene	Unknown	Unknown	Negative	Negative	Negative
Time from diagnosis to RRT (years)	19	18	18	6	2	2
Age at KTx (years)	26	29	26	39	24	53
KTx type	DD	DD	DD	DD	LD	DD
Induction agent	Basiliximab	Thymoglobulin	Thymoglobulin	Basiliximab	Thymoglobulin	Basiliximab
Delayed graft function	No	Yes	No	Yes	No	No
Recurrence timing	1st week	1st week	3 weeks	1st month	1st month	1st week
Post-KTx biopsy IF findings	Unknown	No IF deposits	No IF deposits	C3+	IgM +	IgM 2+ C3+
Initial treatment scheme	PP + CS	PP + RTX	PP + CS	CYC + CS	PP + CS + IvIg	PP + CS + IvIg + RTX + ACTH
Remission	No remission	Complete	Complete	No remission	Complete	No remission
Time to complete remission	-	1 week	2 weeks	-	1 week	-
Outcome	Graft loss 2 months post-KTx	Death with functioning graft (Sudden cardiac death)	Graft loss 58 months post-KTx	Graft loss 2 months post-KTx	Functioning graft (end of follow-up 63 months post-KTx)	Graft loss 7 months post-KTx
Anti-nephrin antibodies in pre-KTx serum measurement	Not measured	Positive (466 UI/mL)	Not measured	Not measured	Not measured	Negative

FSGS: focal segmental glomerulosclerosis; MCD: minimal change disease; RRT: renal replacement therapy; KTx: Kidney Transplant; DD: deceased donor; LD: living donor; PP: plasmapheresis; CS: corticosteroids; RTX: rituximab; CYC: cyclophosphamide; IvIg: Intravenous Immunoglobulin; ACTH: adrenocorticotropic hormone; IF: Immunofluorescence.

We retrospectively assessed anti-nephrin antibodies in pre-transplant serum samples of two patients (supplementary methods). Patient #2 had elevated anti-nephrin antibody titers (466 UI/mL), while Patient #6 had negative titers.

It is unwise to draw broad conclusions from our small cohort, but we can reflect on the observed heterogeneity. As seen with some entities, such as membranoproliferative glomerulonephritis, we believe shifting toward a pathophysiology-based approach could improve stratification, management, and improve future clinical trials. Based on our cohort, we present the following observations:

1. Detection of anti-nephrin antibodies pre-KTx appears to be a key biomarker for predicting the risk of immediate rFSGS. While not universally present, when detected, these antibodies seem to respond well to B-cell depletion agents (and possibly plasma exchange), though the effectiveness in preventing recurrence remains unknown. This was demonstrated in three prospectively followed native-kidney FSGS patients, where rituximab appeared to deplete anti-nephrin autoantibodies and was associated with clinical remission. [3] A recent case report by Habbig et al. also documents successful pre-KTx depletion of anti-nephrin antibodies with rituximab and plasma exchange in a pediatric FSGS patient, resulting in excellent graft function without post-KTx proteinuria. [4] Although not yet confirmed in larger cohorts, in this high-risk patients, preemptive management strategies could be considered to help monitor antibody titers and guide the timing of interventions, potentially preventing or delaying recurrence. The need for standardized, commercially available assays for anti-nephrin antibodies is clear. Such assays would confirm the exact role of anti-nephrin antibodies in prognostication or as therapeutic monitoring tools.
2. Genetic variants do not exclude recurrence. Thousands of genetic variants have been identified across more than 50 podocytopathy-associated genes implicated in FSGS, and most pathogenic variants are associated with a low risk of post-transplant recurrence. As highlighted by Mason et al., many reported cases lack confirmed pathogenicity and true recurrence in monogenic disease appears to be rare [5]. An exception is NPHS1 variants due to alloimmunization against donor nephrin, now considered a distinct entity rather than classical recurrence [5, 6]. Patient #4 carried a heterozygous missense variant in exon 13 of TRPC6 [NM_004621.6:c.2750G>C, p.(Gly917Ala)], classified as a variant of uncertain significance by ACMG guidelines. No familial segregation testing was performed, leaving inheritance undetermined. The variant is not present in databases such as ClinVar or gnomAD. This case illustrates the need for cautious interpretation of variants, ideally with the support of nephrogenetics experts, as variants of uncertain significance with unlikely causality do not rule out FSGS recurrence, and other immune or environmental mechanisms may influence disease expression. Molecular genetic testing remains thus essential in FSGS for predicting post-transplant recurrence risk. In line with this, novel agents are also being developed: a selective TRPC6 inhibitor (BI 764198) is currently undergoing phase 2 clinical trials and may represent a breakthrough in this subset of patients [7]. Gene therapy is also promising, with studies exploring adeno-associated virus-mediated gene delivery, particularly for NPHS2 variants, showing promising results for more targeted treatments [6].
3. There is growing evidence that complement activation plays a role in the pathogenesis of primary FSGS in the native kidney, with studies correlating worse prognosis to low plasma C3 levels, complement deposits in biopsies, and elevated urinary complement byproducts [8]. In our cohort, all patients had histologic features of FSGS in post-transplant biopsies, and C3 deposits were observed in two cases. This suggests that complement activation may reflect more than nonspecific trapping in sclerotic lesions and, despite sample size, still raises the question of whether this early deposition suggests complement-mediated injury or represents an epiphenomenon without a direct correlation to disease activity. Interestingly, Shirai et al. found no complement components colocalizing with nephrin in rFSGS biopsies, despite strong IgG-nephrin interaction [9]. This suggests that anti-nephrin antibody–mediated injury may occur via complement-independent mechanisms, possibly involving non-complement-fixing IgG subclasses. In contrast, detection of C3 deposits in anti-nephrin-negative cases raises the possibility that complement activation may play a larger role in antibody-negative or alternative pathogenic pathways. Of note, in our cohort, the patient with positive anti-nephrin antibodies had no complement deposits, while the seronegative patient did, supporting the idea of distinct pathogenic pathways. Given the growing availability of anti-complement therapies, further research is necessary to determine if complement activation contributes to disease recurrence or works alongside other pathogenic mechanisms, allowing for a more personalized mechanistic-based use of the expanding therapeutic armamentarium.

The multifactorial nature of rFSGS appears to be clear, potentially influenced by genetic variants, circulating permeability factors, and immune-mediated mechanisms. This interaction could explain the variable recurrence rates and outcomes. Further research is necessary to decipher these factors' precise roles and interactions in disease development and progression. Future studies should aim to identify additional circulating permeability factors, explore the role of environmental factors in disease recurrence, and refine genetic testing to predict post-transplant outcomes. Moreover, targeted therapies addressing specific mechanisms—such as B-cell depletion for antibody-related cases, complement inhibition for complement-driven pathology, and genetic therapies for variant-driven cases—may revolutionize treatment and improve long-term graft survival.

## Data Availability

The raw data supporting the conclusions of this article will be made available by the authors, without undue reservation.

## Supplementary Material

SUPPLEMENTARY FIGURE S1 Flowchart showing patient selection for inclusion. A total of 1,372 kidney transplant recipients were retrospectively reviewed. Stepwise exclusions were applied based on native biopsy availability, post-transplant manifestations, and recurrence confirmation by allograft biopsy.
