# Supplementary figures and images for: Beyond Antibodies in Post-Transplant FSGS: New Answers or Recurrent Questions?
Source: Transpl Int. 2025 Sep 10;38:15032. doi: 10.3389/ti.2025.15032 (PMC12457195; doi:10.3389/ti.2025.15032)

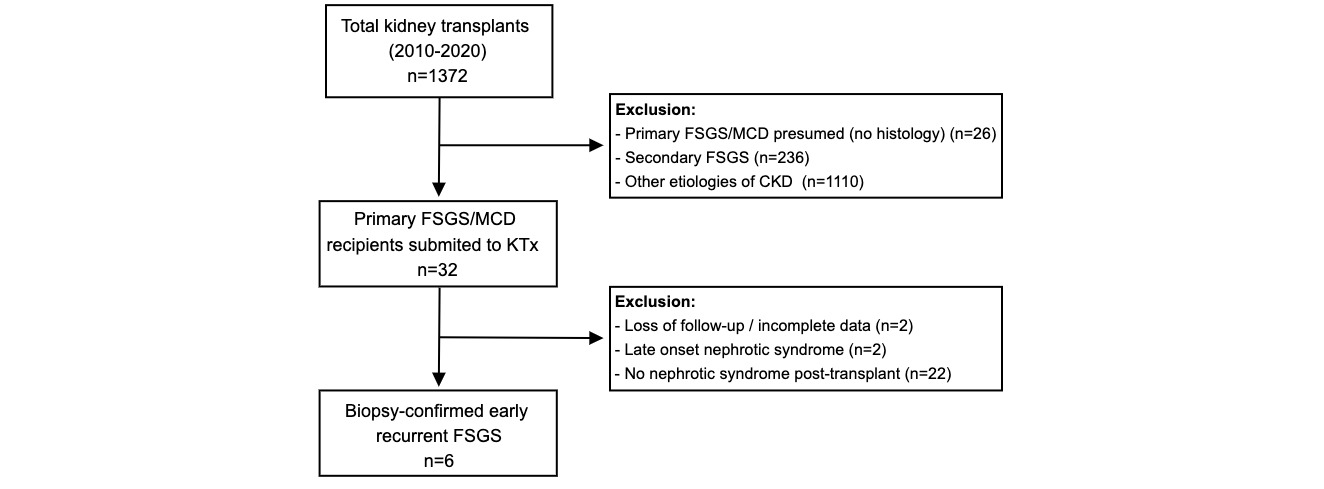

Supplement: Supplementary file 1 [file Image1.jpeg]
