# Supplementary material for: Beyond Antibodies in Post-Transplant FSGS: New Answers or Recurrent Questions?
Source: Transpl Int. 2025 Sep 10;38:15032. doi: 10.3389/ti.2025.15032 (PMC12457195; doi:10.3389/ti.2025.15032)
Supplement: Supplementary file 2 [file DataSheet1.docx]

**Methods - Assessment of Circulating Anti-Nephrin Antibodies**

Anti-nephrin antibodies were assessed at Brigham and Women’s Hospital (Boston, MA) using a previously validated indirect ELISA targeting the extracellular domain of recombinant human nephrin (hNephrinG1059). Sera were collected 4 days before transplantation, stored, and later analysed blinded to clinical outcomes. Individual samples were run in duplicate, and antigen-specific binding was calculated by subtracting the average optical density (OD450 nm) of uncoated wells from that of nephrin-coated wells. A relative antibody titer was determined using a standard curve based on serial dilutions of a positive reference serum assigned a value of 1000 U/mL. A fixed positivity threshold of 187 U/mL was applied, corresponding to the highest titer observed among 30 healthy controls from the Partners Biobank, excluding those with kidney or autoimmune disease. Only two of the six patients in our cohort had available stored serum samples from the pre-transplant period; no samples were available at the time of recurrence due to lack of routine serum storage at our institution earlier in the study period.
